# Valorization of Rose Hip (*Rosa canina*) Puree Co-Product in Enriched Corn Extrudates

**DOI:** 10.3390/foods10112787

**Published:** 2021-11-12

**Authors:** Marta Igual, Maria Simona Chiş, Adriana Păucean, Dan Cristian Vodnar, Sevastița Muste, Simona Man, Javier Martínez-Monzó, Purificación García-Segovia

**Affiliations:** 1 Food Investigation and Innovation Group, Food Technology Department, Universitat Politècnica de València, Camino de Vera s/n, 46022 València, Spain; xmartine@tal.upv.es (J.M.-M.); pugarse@tal.upv.es (P.G.-S.); 2 Department of Food Engineering, Faculty of Food Science and Technology, University of Agricultural Sciences and Veterinary Medicine of Cluj-Napoca, 3–5 Mănăştur Street, 400372 Cluj-Napoca, Romania; simona.chis@usamvcluj.ro (M.S.C.); adriana.paucean@usamvcluj.ro (A.P.); sevastita.muste@usamvcluj.ro (S.M.); simona.man@usamvcluj.ro (S.M.); 3 Institute of Life Sciences, Faculty of Food Science and Technology, University of Agricultural Sciences and Veterinary Medicine Cluj-Napoca, 3–5 Calea Mănăştur, 400372 Cluj-Napoca, Romania; dan.vodnar@usamvcluj.ro

**Keywords:** co-product, *Rosa canina*, snack, extrusion, rose hip

## Abstract

Serious issues and challenges of the world’s population are represented by dwindling natural food resources and the scale-up of sustainable food manufacturing. Therefore, the valorization of co-products from the food industry represents new methods for food development. The principal goal of the study was to capitalize rose hip (*Rosa canina*) co-product powder in extrudates, highlighting its influence on extrusion parameters, physicochemical, and nutritional characteristics. The water absorption index, swelling index, and hygroscopicity increased with the rose hip co-product addition. Furthermore, water solubility index, expansion index, porosity, image parameters (area and perimeter) of the extrudates decreased. Lycopene, β-Carotene, Zea-esters, and lutein were the main carotenoids identified in the extrudates; whereas Catechin, Di-gallic acid, Procyanidin dimmer 1, Procyanidin dimmer 2, and Isorhamnetin-glucuronide were the main flavonoids. Strong Pearson correlations were identified between carotenoids, total flavonoids, vitamin C, total folate, and antioxidant activity. Valorization of the *Rosa canina* powder co-product led to value-added products—corn extrudates—rich in bioactive compounds.

## 1. Introduction

Over the last decade, the emerging trends in food processing have been based on a few pillars, from which the design of health-promoting food and waste reduction is crucial [1,2]. Using this approach, the bio-functional molecules from different unused raw materials, or part of them, could be delivered to the human body, especially when innovative processing technologies are used [3]. This could lead to solving global issues regarding the availability of sufficient food with good nutritional and safety quality and thus assuring the sustainability of food systems [4,5]. With this idea, in our recent publication, we emphasized the possibility to use alfalfa (*Medicago sativa* L.) in corn extrudates manufacturing [6], meantime, Pasqualone et al. [7] highlighted the importance of using legumes in extruded ready-to-eat foods such as lentil [8,9]. In this vein, Herrera-Cazares et al. [10] mentioned that mango (*Mangifera indica* L.) bagasse could be successfully used to manufacture functional confections, whilst microalgae and apple pomace were used in corn extrudates by Uribe-Wandurraga et al. [11] and Karkle et al. [12].

Rose hips, the berry-fruits of many rose bushes species which belongs to *Rosaceae* family, and to the *Rosa* genus with over 150 species, grows in the northern hemisphere, including Europe, the Middle East, America, and Asia [13].

Rose hips (*Rosa canina*) are the only rose hips with documented therapeutic properties, having been used as a medicine for over 2000 years [14].

**Figure 1:**
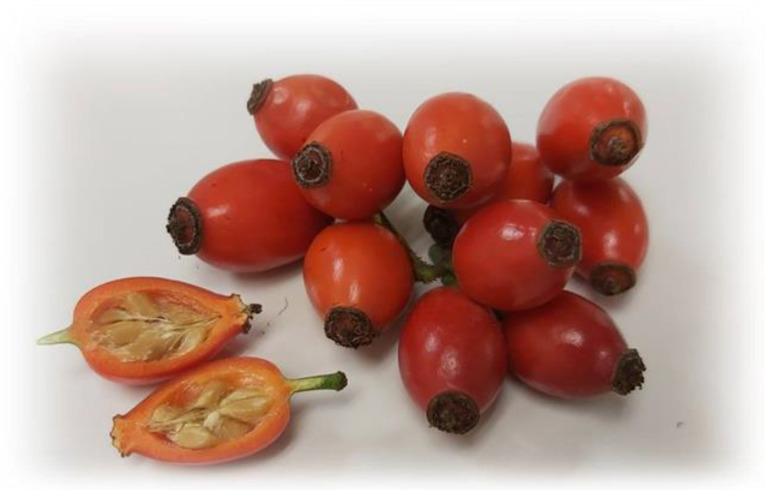
Rose hips of *Rosa canina* and lengthwise section fruit.

*Rosa canina* is an erect shrub up to 3.5 m high. Its branches are often curved or arched, with sharp, hooked prickles, which help the plant climb. The leaves are pinnate, with five to seven leaflets. The flowers of *Rosa canina* are white to pale pink, rarely deep pink, and are a late ripening fruit [14,15]. The pseudo-fruits, rose hips, are aggregate fruits comprising several achenes enclosed by an enlarged, red, fleshy floral cup (hypanthium) (Figure 1). The medium weight of the fruit is 2.8 to 2.9 g, distributed between the pericarp (65–70%) and the seeds (30–35%). The hairy seeds of rose hips fruits contain oil with high linoleic and linolenic acids, carotenoids, and retinol content [13,16,17].

A large body of literature highlighted that rose hips are high in vitamins C, B, D, A, and E, as well as minerals (Mg, Ca, Mn, S, Si, K, Fe, and Se), phenolic compounds, and carotenoids [18,19,20,21,22,23]. They also contain organic acids, pectin, sugars, amino acids, and essential oils [24,25]. The health benefits of rose hip can be primarily attributed to their high concentration of natural antioxidants such as phenolic compounds (tannins, flavonoids, phenolic acids, anthocyanins, and dihydrochalcones), vitamin C (stated to be the richest fruit in vitamin C from all the popular ones), and carotenoids (zeaxanthin, lutein, lycopene, and β-carotene [23,26,27].

Rose hips are widely consumed in countries such as Poland, Finland, Germany, Romania, and Sweden, being an important source of food, medicines, and natural remedies. Common food obtained from rose hips are juice, wine, tea, jelly, marmalade, and syrup but are also used in other types of products such as cookies, cakes, bread, ice cream, pudding, custard soup, pie, beverages, probiotic drinks, and yogurts [13,28]. Furthermore, being rich in vitamin C, rose hip powder is successfully used as a health supplement [29]. Ascorbic acid was mentioned by the literature as having a total amount ranging from 727 mg/100 mL to 943 mg/100 mL (Ercsli, 2007) [20], in the meantime, Medveckiene et al. [23], mentioned for rose hip (*Rosa canina*) a total amount of 1574.13 mg/100 g vitamin C, at V ripening stage. Vitamin C has major importance due to its high antioxidant activity protecting the immune system and helping it to fight against infections [23]. On the other side, Al-Yafeai [19], reported vitamin E content in rose hip (*Rosa canina*) pure as having a total amount of 31.4 µmol/100 g and highlighted its importance in cancer prevention, cardiovascular diseases, and atherosclerosis. With respect to minerals content, in our recent study Igual et al. [30], we emphasized that rose hip (*Rosa canina*) is a rich source in macroelements such as: Ca (7367.09 µg/g), K (6785.77 µg/g), Mg (1558.9 µg/g), and microelements Fe (98.36 µg/g) and Mn (127.56 µg/g). Macro elements have a positive influence on the nervous and muscle system, whilst, microelements are important for hormones and the immune system [31].

To date, rose hips are used as raw materials in the fruit processing industry to obtain the mentioned products; and as with the other fruits’ processing, waste management is an important issue. For example, pomace resulting from juice production, which is still a rich source of health-promoting compounds, is used as a fortifying agent in baked goods or for nutraceuticals [32]. However, the internal structure of rose hips generates other co-products with valuable content of bioactive compounds. This is the case of large quantities of the fleshy fruit pulp which remains trapped between the skin and the hairy seeds after processing rose hips for puree, paste, or tea infusions [30]. This co-product might be a good candidate in functional food design after treatments such as controlled drying and grinding.

Nowadays, snacking registered a significant development mainly due to the worldwide changes in lifestyle behaviors [33]. The positive or negative impact of snacking on health is caused by its nutritional content [33]. Recent studies have reported that snacking increased for about 45% of examined categories, and the snacks are characterized by higher energy content and lower nutritional value [33,34]. A solution could be the development of healthy snacks with low-calorie content and a high density of nutrients. These objectives could be met with the snacks’ enrichment using bioactive compounds from functional food matrices, and with starch acting like fibers in the human body because of specific processing such as extrusion. Extrusion is defined as a combined process between pressure and mechanical force during a period of time [9]. It is mainly used to manufacture ready-to-eat products such as expanded snacks or cereal breakfast, breakfast [11], being a more and more popular approach with many advantages over the traditional processing methods [35].

The extrusion could be conducted in single or/and twin-screw extruders which are both complex bioreactors with different cooking zones. Physico-chemical parameters such as structural, mechanical, and functional ones are highly important in extrudates development [9].

Therefore, this study aimed to evaluate the effects of enrichment with 5% and 10% of rose hip co-product (RHCo) powder on nutritive and functional value, physicochemical characteristics, and extrusion parameters of extruded corn snacks.

## 2. Materials and Methods

### 2.1. Standards and Reagents

Standards of folic and ascorbic acids and carotene were bought from Sigma-Aldrich (Steinheim, Germany). The chemical reagents of analytical grade (H_2_O_2_, HNO_3_, chlorogenic acid, rutin, and gallic acid), were supplied by Merk (Darmstadt, Germany)*;* a Millipore Direct-Q UV system was used for water purification.

### 2.2. Raw Materials

Corn grits (CM) were provided by Maicerías Españolas S.L. (València, Spain). Rose hip (*Rosa canina*) fruits were collected by hand in Aldehuela (Teruel, Spain) in October 2020.

### 2.3. Rose Hip (Rosa canina) Powder Processing

**Figure 2:**
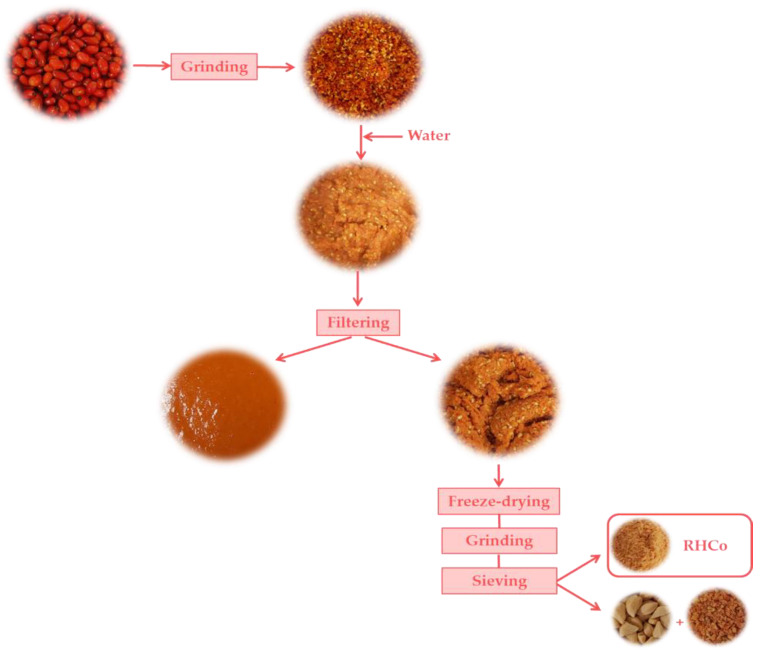
Scheme of rose hips co-product (RHCo) obtaining.

Figure 2 shows a scheme of RHCo powder processing. Rose hips (1 kg) were washed and homogenized with a Thermomix (TM 21, Vorwerk, València, Spain) for 1 min at 5200 rpm. Then, in a 1:1 ratio (rose hip:water), distilled water was added and re-homogenized for 5 min at 5200 rpm. The mixture was filtered using a sieve (light of mesh diameter 1 mm, Cisa 029077). The filtered sample was the rosehip puree, and it was used in our other work [30]. Non-filtered samples were freeze-dried. A non-filtered sample layer was placed in aluminum plates. Samples were frozen at −45 °C (Vertical Freezer, CVF450/45, Ing. Climas, Barcelona, Spain) for 24 h before being dried in a Lioalfa-6 Lyophyliser (Telstar, Spain) at 2600 Pa and −56.5 °C for 48 h. After, samples were ground (Minimoka, Taurus, Lleida, Spain), and was sieved (1 mm, Cisa 029077) to separate RHCo powder from the remains (seeds and other residues).

### 2.4. Formulations and Extrusion Processing

Corn grits were mixed manually using a whisk, with two quantities (5 or 10%; 5RHCo and 10RHCo, respectively) of RHCo to produce the extrusion mixtures (5RHCoM and 10RHCoM).

**Figure 3:**
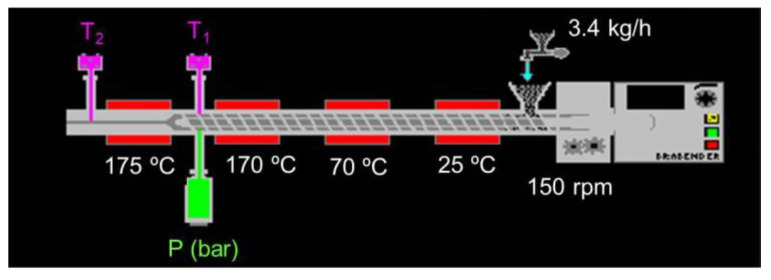
Scheme of conditions used in the extruder.

Figure 3 shows a scheme of the extrusion process performed using a single-screw laboratory extruder (Kompaktextruder KE 19/25; Brabender, Duisburg, Germany) with a barrel diameter of 19 mm and a length:diameter ratio of 25:1. The operating conditions were: 3:1 compression ratio, dosing speed of 18 rpm (feed rate range, 3.4 kg/h), and 3 mm nozzle diameter, as reported in our previous study [6]. The screw was rotated constantly at 150 rpm, and temperatures of barrel sections were set to 25, 70, 170, and 175 °C. Motor torque, screw speed, barrel temperatures (T_1_ and T_2_), and melt pressure (P) were monitored using Extruder Winext software (Brabender). Extrudates were cooled at ambient temperature (23 °C) and sealed in plastic bags for further analysis. Obtained extrudates were CE (control), 5RHCoE, and 10RHCoE.

### 2.5. Analysis

#### 2.5.1. Water Content (x_w_) and Water Activity (a_w_)

Water content is expressed as g water/100 g sample and was determined according to the AOAC for mixtures and extruded samples [36]. The water activity of the extruded samples was determined using the AquaLab PRE LabFerrer equipment (Pullman, WA, USA). Samples were analyzed in triplicate.

#### 2.5.2. Surface Expansion Index (SEI)

The quotient between the square of extrudate diameters and the square of the die diameter determined SEI [37]. The diameter extruded products were measured with an electronic Vernier caliper (Comecta S.A., Barcelona, Spain) 20 times.

#### 2.5.3. Bulk Density (*ρ*_b_) and Porosity (ε)

Bulk density was calculated from the height and diameter of cylinders and their weights (15 times). Porosity is considered the percentage of air volume related to the total volume and was calculated from the real (*ρ*) and bulk densities according to García-Segovia et al. [38] and Equation (1). The real density of the extruded products was determined using a helium pycnometer (AccPyc 1330, Micromeritics, Norcross, GA, USA).

(1) $$\varepsilon =\frac{\left(\rho -\rho_{b}\right)}{\rho }$$

#### 2.5.4. Water Absorption Index (WAI), Water Solubility Index (WSI), and Swelling Index (SWE)

Water absorption index and WSI were determined using the method of Singh and Smith, [39] and calculated according to Uribe-Wandurraga et al. [11] and Equations (2) and (3). A sample was dispersed in distilled water and stirred for 30 min using a magnetic stirrer. Then, they were centrifuged (3000× *g* for 10 min). The supernatant was decanted for determination of its dissolved solids content, and the sediment was weighed.

(2) $$\mathrm{WAI}\;=\frac{\mathrm{weight}\;\mathrm{of}\;\mathrm{sediment}\;}{\mathrm{weight}\;\mathrm{of}\;\mathrm{dry}\;\mathrm{solids}\;}$$

(3) $$\mathrm{WSI}\;\left(\%\right)=\left(\frac{\mathrm{weight}\;\mathrm{of}\;\mathrm{dissolved}\;\mathrm{solids}\;\mathrm{in}\;\mathrm{supernatant}\;}{\mathrm{weight}\;\mathrm{of}\;\mathrm{dry}\;\mathrm{solids}}\right)\times 100$$

The swelling index was measured using the bed volume technique, and the results were recorded and expressed as mm of swollen sample per g of the dry initial sample [40].

#### 2.5.5. Hygroscopicity (Hy)

Extruded samples were placed in an airtight plastic container at 81% relative humidity (Na_2_SO_4_ saturated solution). At the beginning, and after 7 days, samples were weighed, and the Hy was expressed as g of water gained per 100 g dry solids [41].

#### 2.5.6. Texture

A 2 mm diameter cylinder was used with a crosshead speed of 0.6 mm/s for puncture tests using a TA-XT2 Texture Analyzer (Stable Micro Systems Ltd., Godalming, UK); software (Texture Exponent, version 6.1.12.0) registered the force-time curve. The average puncturing force (F_p_), the average specific force of structural ruptures (F_s_), the spatial frequency of structural ruptures (N_sr_), crispness work (W_c_), and the number of peaks (N_o_) were calculated [42,43] according to the following Equations (4)–(6):

(4) $$F_{p}=\frac{S}{t}$$

(5) $$F_{s}=sum\;of\;\frac{F}{N_{o}}$$

(6) $$N_{sr}=\frac{N_{o}}{d}$$

#### 2.5.7. Image Analysis

Digital pictures of each snack were taken immediately after extrusion, using a digital color camera (mod Alpha 330, Sony, Tokyo, Japan). The pictures were taken using a semi-professional kit to control illumination. The digital camera was positioned on a support placed at a fixed distance from the sample, as described by Uribe-Wandurraga et al. [44]. Pictures were taken in triplicate for each sample. The Image J software (ImageJ, NIH, USA) was used to process the images to obtain the area (cm^2^) and perimeter (cm) of samples.

#### 2.5.8. Optical Properties

Translucency and CIE*L*a*b* color coordinates were determined according to Hutchings et al. [45]. Samples were measured on white and black backgrounds considering standard light source D65 and a standard observer 10° (Minolta spectrophotometer CM-3600d, Tokyo, Japan). Measurements of the extruded samples were taken 10 times. Coordinates CIE*L*a*b were used to obtain hue (h*) and chroma (C*) color attributes and the total color differences of mixtures or extrudates with RHCo powder (ΔE_1_) were calculated for the control sample. To evaluate the color changes of the mixtures because of extrusion, the total color difference (ΔE_2_) was calculated between each mixture and extrudate at the same concentrations as the RHCo powder.

#### 2.5.9. Sample Extraction Assisted by Ultrasound

Phenolic compound extraction was described previously by Igual et al. [6]. Briefly, a Reax top (Heidolph, Schwabach, Germany) vortex mixer was used with 0.5 g of sample, 2 mL methanol, and 1% HCl, and after 1 min, the resulted mixture was transferred to an ultrasonic bath (Elmasonic E15H, Elma, Singen, Germany) for 30 min. Next, the samples were centrifuged (4000*× g* for 10 min) using an Eppendorf 5804 centrifuge (Eppendorf, Germany, Hamburg) and filtered by using a 0.45 µm nylon filter (Millipore, Merck KGaA, Darmstadt, Germany). Finally, the resulting sample was inserted into the HPLC system.

For carotenoid extraction, 5 g of each sample was mixed with 5 mL of methanol/ethyl:acetate/petroleum:ether (1:1:1, *v/v*/*v*) and centrifuged (8000*× g* for 5 min) with the same Eppendorf 5804 centrifuge, as described by Szabo et al. [46]. The resulting pellet was subjected to repeated extraction three times, until complete discoloration, while the supernatant was collected in a separation funnel every time and washed with NaCl solution (15%). The organic phase with the targeted carotenoids was dried, with a rotary evaporator (Rotavapor R-124, Buchi, Flawil, Switzerland).

Ultrasound-assisted extraction was used for dehydroascorbic (DHA) and ascorbic acids (AA), as previously showed by Igual et al. [30]. Initially, an aqueous solution containing 0.5 g of sample, 3 mL H_3_PO_4_ with a concentration of 3% and 8% acetic acid were sonicated using Elmasonic E15H (Elma, Singen, Germany) at a temperature of 20 °C, for 30 min. Afterward, the solution was centrifugated for 10 min, at 4 °C, 4000× *g*. The resulted supernatant was filtered and 20 μL were edged into the HPLC system [33].

Folate extraction was previously mentioned by Igual et al. [30]. Shortly, 1 g of sample was blended with 5 mL phosphate buffer (pH = 7), sonicated for 30 min, and centrifuged under the following conditions: 4000× *g* for 10 min and 24 °C.

#### 2.5.10. Analysis of Phenolic Compounds Using HPLC-DAD-ESI-MS

HP-1200 liquid chromatograph (Agilent-Technologies, Santa Clara, CA, USA) with autosampler, MS 6110 single-quadrupole API-electrospray detector and Eclipse XDB-C18 column were used to evaluate phenolic bioactive compounds [6,30].

A mobile phase with a flow rate of 0.5 mL/min composed of acetic acid 0.1% and water (*v/v*) (A) and (B) acetonitrile with acetic acid 0.1% (99:1 *v/v*) were used, as mentioned by Dulf et al. [47]. The detection of phenolics was set at λ = 254, 280, and 340 nm. For MS fragmentation, the ESI (+) module was applied with a scan range of 100–1200 *m/z*, following the same parameters. Agilent ChemStation software (Rev B.04.02 SP1, Palo Alto, CA, USA) was performed for data acquisition.

The identification of phenolic compounds on UV-visible spectra was accomplished by comparing mass spectra with retention time and chromatography with authentic standards (when available). The quantification of flavonoids, hydroxybenzoic acids, and hydroxycinnamic acids was based on the calibration curves of rutin (r^2^ = 0.9972), gallic acid (r^2^ = 0.9978), and chlorogenic acid (r^2^ = 0.9937), respectively. The results were displayed as means ± standard deviations of three repetitions.

#### 2.5.11. Carotenoid Analysis

The protocol described by Szabo et al. [46] was performed for identification, quantification, and separation of carotenoids (reversed-phase EC 250/4.6 Nucleodur 300–5 C-18 ec. column (250 × 4.6 mm, particle size 5 µm; Macherey-Nagel, Germany)). Briefly, acetonitrile:water (9:1, *v/v*) with 0.25% triethylamine (A) and ethyl acetate with 0.25% triethylamine (B) were used as mobile phases, at a flow rate 1 mL/min. The wavelength for chromatograms recording was λ = 450 nm, while the β-Carotene calibration curve (y = 86.781x − 19.028; r^2^ = 0.9931) was applied for carotenoid quantitative determination.

#### 2.5.12. Ascorbic (AA) and Dehydroascorbic (DHAA) Acids Analysis

An HPLC-DAD-ESI-MS system, with an Agilent 1200 HPLC equipment, a quaternary pomp, DAD detector, autosampler, and coupled to an MS-detector single-quadrupole Agilent 6110 (Agilent-Technologies) was used for AA and DHAA analysis, [6]. For separation and identification, an XDB C18 Eclipse column (4.5 × 150 mm, particle size 5 μm) was used. The working parameters were: 100–600 *m/z* scanning range in ESI (+) mode used for MS fragmentation, 3000 V capillary voltage, 300 °C temperature, nitrogen flow of 7 L/min, spectral absorbance of 200–400 nm, and wavelength of λ 240 nm. Agilent ChemStation software (Rev B.04.02 SP1) was used for data analysis.

#### 2.5.13. Folate Determination Using HPLC-DAD-ESI-MS Assay

Folate identification and quantification was performed on the same HPLC-DAD-ESI-MS system as described in Section 2.5.12. The specific parameters for the XDB C18 Eclipse column (4.5 × 150 mm, particle size 5 μm) were: isocratic system, with a flow rate of 0.5 mL/min, a ratio of 20:80 (*v/v*) between acetonitrile:acetic acid 1% and a temperature of 25 ± 0.5 °C, as previously described by Igual et al. [30]. Chromatograms were recorded at wavelength λ = 280 nm and data acquisition was completed using the Agilent ChemStation software (Rev B.04.02 SP1).

#### 2.5.14. Antioxidant Capacity (AC)

Antioxidant capacity was determined using the free radical scavenging activity with DPH (2,2-diphenyl-1-picryl-hydrazyl-hydrate) according to the method of Igual et al. [48] in triplicate. A UV-visible spectrophotometer (UV-3100PC, VWR, Radnor, PA, USA) was used to measure the absorbance at 515 nm. The results were indicated as µg Trolox equivalents (TE) per gram dry weight (µg TE/g d.w.).

### 2.6. Statistical Analysis

Analysis of variance (ANOVA), with a confidence level of 95% (*p <* 0.05), using Statgraphics Centurion XVII Software, version 17.2.04 (Statgraphics Technologies Inc. The Plains, VA, USA), was applied to evaluate the differences among mixtures or extruded samples, and to evaluate the extrusion process. A correlation analysis among the extrusion parameters and textural properties of produced extrudates, with a 95% significance level, was conducted (Statgraphics Centurion XVII). The linear correlation between AC and the analyzed compounds was explained by the Pearson correlation coefficient. All measurements were made in triplicate.

## 3. Results and Discussion

### 3.1. Parameters of Extrusion Process

Table 1 shows process control parameters. Melt pressure (P) and barrel temperatures (T_1_ and T_2_) were monitored during extrusion. Specific mechanical energy (SME) was calculated according to Logié et al. [49] and Equation (7):

(7) $$SME=\frac{C\times V}{Q}$$

where *C* is the torque (N m), *V* is the screw speed (rad s^−1^), and *Q* is the mass flow rate (g s^−1^). *SME* can be defined as the energy required for the production of 1 g of extrudate [50]. Moisture content, particle size, and composition of the material used for extrusion are factors that directly affect SME. Specific mechanical energy can also evaluate the suitability of extruder machines for product development [51]. Table 1 also includes water loss (W_L_) during the extrusion process, calculated from the water content of the feed and extrudates mixtures. Water loss at the die depends on the vapor pressure inside the air cells and matrix characteristics, such as extensibility and water binding. It reflects starch transformation; ungelatinized starch presence reduces extensibility, and residual water is trapped inside the structure rather than escaping at the vapor flashpoint [52]. The pressure measured on the extruder head ranged between 107 and 155 bar. Similar values were obtained for corn extrusion in other studies about the inclusion of house cricket powder and lucerne power in mixtures [6,43], where *p* ranged from 140 to 190 and from 112 to 222, respectively. The addition of RHCo in mixtures increased *p* significantly during extrusion (*p <* 0.05), reaching higher values when higher RHCo content was in mixtures. The pressure generally increases with decreasing moisture content [6]. In this study, the water content of CM (9.17 g_w_/100 g) was significantly (*p <* 0.05) higher than 5RHCoE (8.58 g_w_/100 g) or 10RHCoE (8.06 g_w_/100 g). This was reflected in a significant (*p <* 0.05) lower pressure during extrusion in CE. Furthermore, T_2_ increased as the% RHCo increased. SME ranged from 756 to 925 J/g, like other studies about the inclusion of house cricket powder and lucerne power in mixtures [6,43] with values of 900–1100 and 800–1000 J/g, respectively. Mixtures enriched with RHCo required significantly (*p <* 0.05) lower SME for extrusion than control samples. Furthermore, these samples lost significantly (*p <* 0.05) higher water content than the control. Probably, in samples with RHCo incorporation, there is a greater amount of water to be absorbed by the fiber of RHCo and, consequently, greater will be the loss of water at the open of the die with the difference in pressure. Therefore, the expansion will be smaller, as besides the higher W_L_, insoluble fibers are also biopolymers which are already slightly extendable, unlike starch [52].

### 3.2. Physicochemical Characteristics of Extrudates

The physicochemical properties of extrudates are direct indicators of the effect on product quality from raw material and processing variables. Table 2 shows extrudates’ mean values and standard deviations of x_w_, a_w_, WAI, WSI, SWE, Hy, SEI, *ρ_b_*, and *ε*. Water content decreased due to RHCo addition, however, there were only significant differences between CE and 5RHCoE. The control extrudate presented 4.8 (0.3) g_w_/100 g, like other studies of corn extrudates [11,43]. There were no significant a_w_ differences (*p* > 0.05). The values of a_w_ were like other corn snacks obtained by García-Segovia et al. and Igual et al. [6,38], who presented values ranging from 0.346 to 0.366 for corn extrudates. RHCo enrichment significantly increased WAI, SWE, and Hy (*p* < 0.05) but provoked a significant decrease in WSI and *ε*. Moreover, this behavior was more intensive when higher RHCo concentration was in mixtures.

The water absorption index and WSI show how extrudates interact with water [53]. WAI indicates the portion of water absorbed by the extrudate when immersed in water [54], whereas WSI indicates the water solubilized components released during extrusion that can cause molecular damage [55]. In Table 2, CE showed the lowest WAI values, however, it showed the highest values of WSI. Samples with RHCo presented WSI values lower than CE. Thus, according to WSI values, extrudates with RHCo in mixtures could be more stable samples due to the reduction of molecular damage. The SWE mean values expressed as mL_swollen_/g_dry solid_ are also shown in Table 2. Samples 10RHCoE significantly presented the highest values of SWE (*p* < 0.05), whereas the control SWE was like other studies [26,33], which presented values ranging from 2.38 to 2.48 mL swollen/gdry solid for corn extrudates. Furthermore, adding RHCo in this study increased Hy.

Typical extrudate structures are due to the sudden expansion—at the exit—of the molten mass from the restricted die, from high pressure to atmospheric pressure [42]. Adding RHCo to extrusion mixtures decreased the SEI values (Table 2). This expansion loss was also observed in other studies when house cricket powder and lucerne power were added to obtain corn extrudate [6,43]. During extrusion, fibers create a network that affects the distribution of water in the matrix, modifying the extension characteristics because fiber and starch compete for water and leads to a delay in gelatinization of starch, thus, reduced expansion [56].

Table 2 also includes *ρ_b_* and *ε* of extrudates. Density is a general property of the extrudate, which indicates changes in material parameters plus cell structure, pores, and voids developed as the results of the processing; highly expanded extruded materials show a porous structure [51] which is measured by *ε*. There were significant (*p <* 0.05) differences in *ε* values between control and enriched samples, with the values of the control being higher, whereas ε decreased as the% RHCo increased.

Mean values of W_c_, N_sr_, F_s_, F_p_, and N_0_ of extrudates from texture analysis are shown in Table 3; furthermore, A and P from image analysis are also shown in Table 3. Texture is one of the most important characteristics of extruded snacks that help improve the quality of food products [57]. Crispiness work can be interpreted as the sensory parameter of fracturability and describes the work required to fracture one pore or a group of pores. Puncturing force and F_s_ of extruded products are usually associated with the sensory perception of hardness during chewing; defined as the force to compress a solid substance between the molar teeth [51]. The spatial frequency of structural ruptures describes the number of fracture events during puncture, and N_0_ is the number of fractures along with the puncture assay. The control extrudate presented more fracture resistance and was harder than the rest, however, the extrudates enriched with RHCo were crisper, especially the 5RHCoE sample that showed significant differences in N_0_ (*p* < 0.05). Probably it was related to the presence of fiber in RHCo and its behavior during extrusion explained in Section 3.1.

**Figure 4:**
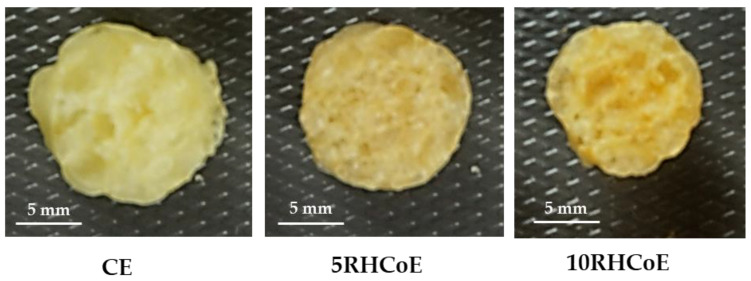
Radial cross-sections of extruded samples obtained from image analysis.

Figure 4 shows radial cross-sections of extruded samples obtained from image analysis. The A and P (Table 3) decreased significantly by RHCo addition (*p* < 0.05). The A was significantly affected by RHCo concentration in mixtures (*p* < 0.05), where higher% RHCo decreased A values, like with SEI values (Table 2), likely due to the higher W_L_ and insoluble fibers of RHCo, that are biopolymers which are slightly extendable, unlike starch [52].

Pearson correlation coefficients among studied parameters and RHCo percentages were obtained (Table 4). There were significant (*p <* 0.05) correlations between RHCo percentages and WAI, WSI, SWE, Hy, SEI, *ε*, A, and P. When RHCo% increased, WAI, SWE, and Hy increased, whereas WSI, SEI, *ε*, A, and P decreased. These correlations were also shown in studies with corn extrudates enriched with lucerne [6]. Therefore, the greater RHCo addition, the longer the shelf life of the product and the lower the risk of molecular damage, but its expansion will be less. The SWE presented the highest Pearson coefficient with an increase RHCo%, probably due to the high fiber content of the co-product that caused greater swelling of extrudates when in contact with water. The image parameters (A and P) presented higher significant correlations with SWE, SEI, and *ε*. As observed in other works, [38], W_c_ was significant and positively correlated with x_w_ because extrudates with higher water content showed more resistance to fracture. Porosity was correlated significantly and positively with WSI and SEI, and negatively with WAI, SWE, and Hy as it was shown in other corn extrudates enriched with lucerne [6]. Expansion properties correlated with each other, high SEI leads to high *ε* and low *ρ_b_*, and vice versa, which corroborates with previous studies such as García-Segovia et al., and Agathian et al. [38,58].

**Figure 5:**
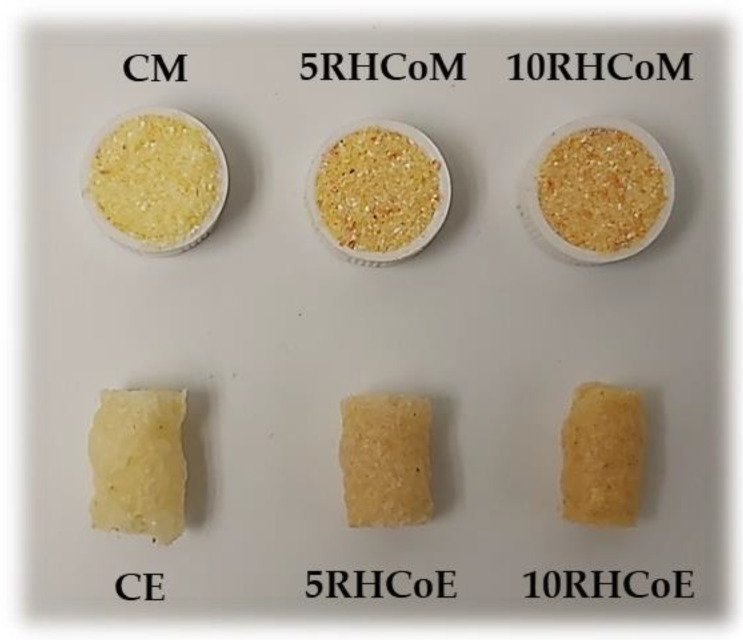
Appearance of studied mixtures (M) and extrudates (E); C: Control, 5RHCo: 5% of rosehip co-product and 10RHCo: 10% of rosehip co-product.

Color parameters L*, a*, b*, C*, h*, and ΔE of mixtures and extrudates are included in Table 5. Mixtures and extrudates were not translucent because there are no differences in the color measurements taken on white and black backgrounds of both mixtures and extrudates with or without RHCo. Therefore, color coordinates CIE*L*a*b* and the values of C* and h* were obtained directly from the equipment used for color measurement, like in other studies [30]. RHCo addition mixtures significantly decreased (*p <* 0.05) L* and h* but significantly increased (*p <* 0.05) a*. All mixtures were redder with increased addition of RHCo, and were significantly (*p <* 0.05) redder than CE. Color parameters L*, a*, b*, and C* decreased significantly (*p <* 0.05) due to the extrusion process. As observed in Figure 5, 10RHCoM and 10RHCoE were the samples redder in accordance with the highest values of a*. Total color differences between samples with RHCo and the control (ΔE_1_) ranged between 4.4 and 13.5, higher than three units; therefore, humanly perceptible [59]. Color is an important quality parameter because it reflects the extent of chemical reactions and cooking or degradation that takes place during the extrusion process. According to Dogan et al. [60] extrusion provokes darker products with a more intense yellow color. In this study, ΔE_2_ ranged between 24 and 32.21. These ΔE_2_ values were significantly higher in CE and lower in 10RHCoE (*p* < 0.05).

Figure 5 shows the appearance of the mixtures and extrudates. In concordance with the color coordinates, the reddish color (a* increase) of the mixture with higher RHCo addition is remarkable. In the same sense that ΔE_2_ marked (Table 5), extrudates lost the reddish color compared to mixtures.

### 3.3. Nutritional and Functional Value of Mixtures and Extrudates

#### 3.3.1. Phenolic Acid Content of Mixtures and Extrudates

In this study, RHCo did not exhibit a positive influence on mixtures and extrudates’ phenolic acid contents (Table 6) as expected, however, corn flour was richer in phenolic compounds [30]. This could be explained by rose hip being rich in chlorogenic acids [61], compounds not identified in this study. However, Medveckienė et al. [23] highlighted phenolic composition accumulation could be also influenced by some enzymes and genes whose activity could vary between species and genotypes.

Related to the extrudates phenolic acid content (Table 6), the extrusion process negatively influenced the p-coumaric and syringic acids content, whereas ferulic and Di-caffeic acids increased their value after extrusion. Likewise, Pasqualone et al. [7] emphasized extrusion could have a detrimental influence on pea/rice and starch/navy bean extrudates, whereas, phenolic extrudates manufactured with pea, rice, and carob flour increased their content through extrusion.

The decrease of total phenolic content due to the thermal treatment was also stated by Kadakal and Duman [62] who showed a thermal degradation of rutin and total phenolic compounds during nectar manufacturing using rose hip. A temperature higher than 70 °C caused a higher degradation rate of the compounds. In contrast, Pasqualone et al. [7] found extrusion could increase the phenolic amount through the inactivation of oxidative enzymes, mainly responsible for their degradation. For instance, in the present study, p-coumaric and syringic CE sample acids contents were 59.81 and 12.32 μg/gd.w. and after 10% RHCo addition the extrudates reached values of 55.8 and 11.41 μg/gd.w., respectively. On the other hand, ferulic and Di-caffeic extrudates acids increased their content through RHCo addition reaching final values 36.58 and 51.7 μg/gd.w., respectively. Polyphenols could help prevent different human neurogenerative diseases and cardiovascular disorders, having a high antioxidant activity and exhibiting antimicrobial effects [63].

The hydroxybenzoic acid (Di-Gallic acid) and flavonols content of mixtures and extrudates are displayed in Table 7. Considerably higher content of flavonoids was identified in RHCo, with the main components: Di-gall (4204.43 µg/g d.w.), Cat (1348.67 µg/g d.w.), Procyand 2 (937.64 µg/g d.w.), Procyand 1 (775.92 µg/g d.w.), Q-gluc (235.73 µg/g d.w.), and Q-glu-gluc-rham (190/55 µg/g d.w.), (data not shown). The rich total flavonoid content (0.43 g/100 g d.w.) of rose hip pomace obtained through fresh juice manufacturing was highlighted also by Tańska et al. [32]. Likewise, Medveckienė et al. [23] mentioned that flesh flavonoids content from different rose hips could range between 52 and 56 mg/100 g d.w., but in their study, the amount varied between 34.23–41.59 mg/100 g d.w. Flavonoids are important bioactive compounds with multiple biological functions such as being antioxidative, helping to prevent ischemic stroke, kidney stones, hepatoprotective, being an anti-depressant, and helping cardiovascular biological function [16].

The addition of RHCo in mixtures increased the flavonols content mainly because of RHCo important flavonoids content, considering that CM content in flavonols was not detected in this study (Table 7). Catechin (361.2 μg/gd.w.), Di-Gallic acid (193.2 μg/gd.w.), Procyanidin dimmer 2 (169.4 μg/gd.w.), and Procyanidin dimmer 1 (143.1 μg/gd.w.) were the main flavonols identified in 10RHCo mixtures. The result is in line with our recent study Igual et al. [30] where we showed that the addition of 8% of rose hip (*Rosa canina*) powder increased flavonols content mixtures, reaching values of 737.79 μg/gd.w., 148.0, and 205.2 μg/gd.w for Di-Gallic acid, Procyanidin dimmers 1 and 2.

Regarding extrudates content, the extrusion process decreased the content of flavonols and Di-gallic acid, as we have previously shown [30]. Briefly, in our previous study, the extrusion process of corn extrudates enriched with 8% rose hip (*Rosa canina*) powder decreased by 3.40 times the total flavonoids amount.

Total flavonoids content identified in the present study for 5RHCoM and 10RHCoM samples, were 503.0 μg/gd.w. and 975.0 μg/gd.w, respectively. The extrusion process decreased their amounts to the final values of 280.0 and 694 μg/g d.w. For instance, Di-Gallic acid (Di-Gall) content of the 10RHCoE sample decreased during the extrusion process by 14%, whilst, quercetin-glucoside (Q-gluc) decreased its value by 23.84%. In the same line, using 8% rose hip (*Rosa canina*) powder addition in corn extrudates, Igual et al. [30] showed that Di-Gall decreased its value by 53% meanwhile, Q-gluc decreased through extrusion by 25.44%, respectively.

#### 3.3.2. Carotenoid Content of Mixtures and Extrudates

Carotenoids are defined as bioactive compounds involved in the color of the plants and fruits, being divided into two main subgroups: xanthophylls (mainly lutein, cryptoxanthin, and zeaxanthin) and carotenes (lycopene and β-carotene). For example, xanthophylls are responsible for red color, whereas carotenes are involved in the red and/or orange color of plants and fruits [14]. Lycopene is characterized by Volker et al. [64] as an acyclic carotenoid with the ability to act as a strong antioxidant and was identified in rose hip in a range of 9.07–19.93 mg/100 g. Medveckiene et al. [23] identified lutein, zeaxanthin, lycopene, and β-Carotene in the following amounts: 1.55, 0.23, 2.14, and 3.95 mg/100 g d.w, respectively. However, rose hip shells (red pseudo-fruit flesh) were claimed as a rich source of carotenoids [65].

In this study, RHCo is a rich source of carotenoids, from which lycopene (80.27 µg/g d.w), β-carotene (66.50 µg/g d.w), Zea-ester (35.77 µg/g d.w), Lut-ester (16.68 µg/g d.w), and lutein (6.22 µg/g d.w) achieved the highest extended values. Likewise, Medveckienė et al. [23] showed rose hip flesh is high in total carotenoids, from which β-Carotene ranged between 45.56–70.34%, lutein and zeaxanthin represented 12.89–20.53%, and lycopene 9.29–24.68%. The differences in carotenoid content could be justified by several factors, such as the degree of fruit ripening and its maturity, genetic factors, climate, storage conditions, and method of extraction [23]. Furthermore, recently, Oprică and Roșu [66] showed that *Rosa canina* flavonoids content could be positively influenced by altitude.

Increasing RHCo in mixtures positively influenced carotenoid amounts, mainly of lycopene, β-Carotene, and Zea-ester, as highlighted in Table 8. The mixture’s color coordinates (mainly a* which is a red/green coordinate) increased its value with RHCo addition (Table 5), probably due to its carotenoid content.

The extrusion process decreased the extrudates carotenoids content (Table 8) but lycopene, β Carotene, and Zea-ester remained identifiable. The decrease of carotenoid content during the extrusion process could be explained by their instability to light, temperature, and oxygen, being defined as unstable and sensitive molecules [67]. The idea of decreasing carotenoid content through extrusion was recently reinforced by Paznocht et al. [68] who showed that lutein, β and α carotene, zeaxanthin, xanthophyll esters, and antheraxanthin decreased to 25.7% by extrusion process. During extrusion, a* decreased, mainly because the carotenoids amount decreased due to the thermal treatment.

#### 3.3.3. Ascorbic and Dehydroascorbic Acids, Vitamin C, Folate, and Antioxidant Activity

According to Drozdz et al. [69] among wild and cultivated trees and shrubs, rose hip fruits represent the richest natural source of AA ranging between 600 and 1000 mg/100 g and mainly identified in the pomace. In this study, the AA content was 3274.79 µg/g d.w, whereas vitamin C content was 4427.81 µg/g d.w. Medveckienė et al. [23] reported a value of vitamin C for rose hip (*Rosa canina*) fruits in the V ripening stage of 1574 mg/100 g and mentioned that the vitamin C amount is related to several factors such as genotypic factors, climatic conditions, harvesting period, maturity fruit stage, and postharvest manipulations. Likewise, Oprica and Roșu [66] showed rose hip contained a vitamin C amount between 30 and 1300 mg/100 g, being one of the highest values among vegetables and fruits; furthermore, Fascella et al. [70] found a total AA amount of 513.95 mg/100 g d.w. in *R. canina* fruits from Sicily.

The RHCo folates content was 290.97 µg/g d.w., whereas the AA reached a higher extended value: 12,567 µg TE/g d.w. (data not shown). The high AA level was also mentioned by Fascella et al. [69] who emphasized *Rosa canina* with a total antioxidant activity of 4493.64 μmol TE/g d.w. In our previous study [30] we identified a total amount of 306 µg/g rose hip folates, whereas the AA was 19.23 mg TE/g.

The mixtures and corn extrudates vitamin C, folates, and AC content are highlighted in Table 9. Extrusion processing exhibited a detrimental effect on these bioactive compounds. The decrease in vitamin C during thermal treatment was emphasized by Gulati et al. who mentioned the instability of vitamin C against oxidation and heat [71]. Likewise, we identified extrusion processing reduced vitamin C, folates, and AC contents [30].

The daily recommended folate allowance is 200 µg, according to the Council directive published on 24th September 1990 [72]. According to Regulation no.1924/2006 of the European Parliament and of the Council (20 December 2006) on nutrition and health claims made on foods [73], extrudates manufactured with RHCo could be a source of folate. Therefore, the consumption of 13.5 and 20 g of 10RHCoE and 5RHCoE, respectively, could ensure the recommended daily folate consumption. Furthermore, it is important to mention the extrudates vitamin C content, which has a recommended allowance of 60 mg/day. Therefore, the 10RHCoE sample could be considered a source of vitamin C, with a total amount of 456.68 µg/g.

Pearson correlation analyses were used to explain the relationship between vitamin C, AA, folates, total phenolic acids, total flavonoids, and total carotenoids with AC. All studied compounds showed a positive Pearson’s correlation coefficient with AC, except total phenolics acids that showed a negative coefficient. Total carotenoids played a significant role in the AC of mixtures and extrudates (0.9840, *p <* 0.05), followed by total flavonoids (0.8747, *p <* 0.05) and vitamin C (0.8049, *p <* 0.05). Likewise, Fascella et al. [70] showed a strong relationship between carotenoids, flavonoids, and vitamin C contents in rose hip, because flavonoids and carotenoids could prevent vitamin C oxidation. Studies have also observed a high correlation between flavonoids and AC in grapefruit powders [74]. Furthermore, authors have reported the main contributing AC factor was vitamin C or AA [48,75].

## 4. Conclusions

Because consumer attention is focused on dietary supplements, natural sources rich in bioactive compounds are thrown away as waste. In this study, the addition of 5% and 10% of RHCo enriched extrudates with flavonols, carotenoids, vitamin C, folate, and antioxidant activity. For instance, phenolic acids increased from a value of 165.63 μg/g dry weight for the control sample to a final value of 173.3 μg/g dry weight for 10RHCoE, whilst total flavonols 10 RHCoE samples were enriched to higher extended values (694.0 μg/g dry weight). Vitamin C had a value of 56.19 μg/g dry weight for control samples and increased its value eightfold times by 10% addition of RHCoE, meantime, antioxidant activity increased threefold times.

Moreover, the physicochemical characteristics of extrudates were improved by RHCo addition, from which the risk of molecular damage, measured through WSI and SWE, exhibited higher extended values.

This study demonstrated that RHCo could be successfully used in corn extrudates to improve their nutritional value. This study aids the utilization of rose hip co-products in ready-to-eat food production.

## Figures and Tables

**Table 1:** Melt pressure (*p*), barrel temperatures (T_1_, T_2_), specific mechanical energy (SME), and water loss (W_L_) of studied samples. Mean values ± standard deviation.

Sample	CE	5RHCoE	10RHCoE
P (Pa)	107 ± 4 ^c^	123 ± 7 ^b^	155 ± 5 ^a^
T_1_ (°C)	184.3 ± 0.9 ^b^	186.1 ± 0.4 ^a^	185.8 ± 0.7 ^a^
T_2_ (°C)	76.25 ± 1.04 ^c^	80.4 ± 0.5 ^b^	83.0 ± 1.4 ^a^
SME (J/g)	925 ± 6 ^a^	756 ± 5 ^b^	761 ± 4 ^b^
W_L_ (g_w_/g_db_)	0.1432 ± 0.0007 ^c^	0.243 ± 0.003 ^a^	0.1915 ± 0.0006 ^b^

Different small letter in superscript within row indicates significant differences between samples (*p* < 0.05). CE: Control extrudates; 5RHCoE: Extrudate with 5% of rosehip co-product and 10RHCoE: Extrudate with 10% of rosehip co-product.

**Table 2:** Water content (x_w_), water activity (a_w_), water absorption index (WAI), water solubility index (WSI), swelling index (SWE), hygroscopicity (Hy), expansion index (SEI), bulk density (*ρ_b_*) and porosity (*ε*) of extrudates. Mean values ± standard deviations.

Sample	CE	5RHCoE	10RHCoE
x_w_ (g_w_/100 g)	4.8 ± 0.3 ^a^	3.67 ± 0.07 ^b^	4.0 ± 0.3 ^ab^
a_w_	0.366 ± 0.003 ^a^	0.362 ± 0.003 ^a^	0.367 ± 0.003 ^a^
WAI	3.921 ± 0.015 ^c^	4.95 ± 0.03 ^b^	5.298 ± 0.003 ^a^
WSI (%)	18.8 ± 0.2 ^a^	11.8 ± 0.2 ^b^	9.86 ± 0.03 ^c^
SWE (mL_swollen_/g_dry solid_)	2.323 ± 0.003 ^c^	3.06 ± 0.02 ^b^	3.78 ± 0.09 ^a^
Hy (g_w_/100 g_dry solid_)	19.52 ± 0.16 ^c^	21.66 ± 0.12 ^b^	24.3920 ± 0.0005 ^a^
SEI	14.6 ± 0.3 ^a^	13.8 ± 0.3 ^b^	10.2 ± 0.3 ^c^
*ρ*_b_(g/cm^3^)	0.092 ± 0.008 ^b^	0.076 ± 0.002 ^b^	0.128 ± 0.007 ^a^
ε (%)	93.0 ± 0.6 ^a^	92.3 ± 0.2 ^b^	88.6 ± 0.6 ^c^

Different small letter in superscript within row indicates significant differences between samples (*p* < 0.05). CE: Control extrudates; 5RHCoE: Extrudate with 5% of rosehip co-product and 10RHCoE: Extrudate with 10% of rosehip co-product.

**Table 3:** **C**rispness work (W_c_), spatial frequency of structural ruptures (N_sr_), average specific force of structural ruptures (F_s_), average puncturing force (F_p_), and number of peaks (N_0_) of extrudates from texture analysis, area (A) and perimeter (P) from image analysis. Mean values ± standard deviations.

Sample	CE	5RHCoE	10RHCoE
W_c_ (N·mm)	0.26 ± 0.3 ^a^	0.14 ± 0.02 ^b^	0.17 ± 0.03 ^b^
N_sr_ (mm^−1^)	9.69 ± 1.05 ^b^	11.7 ± 0.7 ^a^	12.3 ± 0.7 ^a^
F_s_ (N)	2.5 ± 0.3 ^a^	1.7 ± 0. 2 ^b^	2.1 ± 0.4 ^b^
F_p_ (N)	2.0 ± 0.3 ^a^	1.4 ± 0.2 ^b^	2.1 ± 0.2 ^a^
N_0_	108 ± 8 ^b^	128 ± 8 ^a^	111 ± 11 ^b^
A (cm^2^)	1.38 ± 0.12 ^a^	1.26 ± 0.08 ^b^	1.10 ± 0.05 ^c^
P (cm)	4.4 ± 0.2 ^a^	4.18 ± 0.17 ^a^	3.98 ± 0.13 ^b^

Different small letter in superscript within row indicates significant differences between samples (*p* < 0.05). CE: Control extrudates; 5RHCoE: Extrudate with 5% of rosehip co-product and 10RHCoE: Extrudate with 10% of rosehip co-product.

**Table 4:** Pearson correlation coefficients among studied parameters of extruded product and RHCo percentage.

	a_w_	WAI	WSI	SWE	Hy	SEI	*ρ* *_b_*	*ε*	W_c_	N_sr_	F_s_	F_p_	N_0_	A	P	%RHCo
x_w_	0.1927	−0.8015	0.8105	−0.6237	−0.8090	0.3603	0.0817	0.7390	0.8812 *	−0.7273	0.7064	0.4294	−0.4465	0.5368	0.4983	−0.6369
a_w_		−0.067	0.1092	0.1169	−0.0581	−0.3807	0.5965	0.0365	0.4438	−0.7483	0.2457	−0.1351	−0.7971	−0.2867	0.3144	0.1387
WAI			0.8368 *	0.9606 *	0.9989 *	−0.8085	0.4330	−0.9902 *	−0.4646	0.4571	−0.2208	0.0782	0.0124	−0.8834 *	−0.8395 *	0.9618 *
WSI				0.9500 *	−0.9963 *	0.0515	−0.4034	0.9896 *	0.4868	−0.4939	0.2357	−0.0731	−0.0552	0.8669 *	0.8245 *	−0.9506 *
SWE					0.9583 *	0.0632	0.6439	−0.9740 *	−0.2092	0.2074	−0.0040	0.2234	−0.2544	−0.9619 *	−0.9169 *	0.9981 *
Hy						−0.8038	0.4251	−0.9838 *	−0.4689	0.4510	−0.2348	0.0681	0.0061	−0.8815 *	−0.8392 *	0.9596 *
SEI							−0.8695 *	0.8496 *	0.3482	0.1018	−0.2802	−0.3690	0.5446	0.9352 *	0.8685 *	−0.9385 *
*ρ*_b_								−0.5022	0.5098	−0.4047	0.6614	0.5702	−0.7820	−0.6640	−0.5652	0.6567
ε									0.3916	−0.4463	0.1394	−0.1278	0.0409	0.9091 *	0.8665 *	−0.9736 *
W_c_										−0.8703 *	0.8634 *	0.6123	−0.7892	0.1088	0.0965	−0.2213
N_sr_											−0.5540	−0.1901	0.8834 *	−0.0109	0.0154	0.2167
F_s_												0.8719 *	−0.6164	0.0280	0.0869	−0.0006
F_p_													−0.3712	−0.1059	−0.0662	0.2184
N_0_														0.4037	0.3896	−0.2502
A															0.9802 *	−0.9531 *
P																−0.8968 *

Water content (x_w_), water activity (a_w_), water absorption index (WAI), water solubility index (WSI), swelling index (SWE), hygroscopicity (Hy), expansion index (SEI), bulk density (*ρ*_b_), porosity (*ε*), crispness work (W_c_), spatial frequency of structural ruptures (N_sr_), average specific force of structural ruptures (F_s_), average puncturing force (F_p_), and number of peaks (N_0_), A (area), P (perimeter) of extrudates. * Correlation is significant at 0.05.

**Table 5:** Color coordinates (L*, a*, b*, C*, and h*) and total color differences (ΔE) of corn mixtures and extrudates. Mean values ± standard deviations.

	Mixtures			Extrudates		
	CM	5RHCoM	10RHCoM	CE	5RHCoE	10RHCoE
L*	80.5 ± 0.5 ^aA^	72.06 ± 1.12 ^bA^	72.0 ± 1.7 ^bA^	60.3 ± 0.9 ^aB^	54.2 ± 0.3 ^bB^	53 ± 2 ^bB^
a*	6.3 ± 0.7 ^bA^	13.3 ± 0.6 ^aA^	14.7 ± 1.3 ^aA^	0.3 ± 0.4 ^cB^	5.52 ± 0.15 ^bB^	10.938 ± 1.095 ^aB^
b*	41.5 ± 1.7 ^aA^	42.2 ± 0.2 ^aA^	38.5 ± 0.3 ^bA^	17.1 ± 0.7 ^cB^	21.8 ± 0.2 ^bB^	26 ± 2 ^aB^
C	41.9 ± 1.8 ^bA^	44.3 ± 0.4 ^bA^	41.2 ± 0.4 ^aA^	17.1 ± 0.7 ^cB^	22.5 ± 0.2 ^bB^	28 ± 2 ^aB^
h	81.4 ± 0.6 ^aB^	72.5 ± 0.7 ^bB^	69.1 ± 1.7 ^cA^	89.0 ± 1.2 ^aA^	75.8 ± 0.3 ^bA^	67.0 ± 1.2 ^cB^
ΔE_1_	-	11.0 ± 1.3 ^bA^	28.3 ± 0.2 ^aA^	-	9.3 ± 0.3 ^bB^	16 ± 2 ^aB^
ΔE_2_	-	-	-	32.21 ± 0.12 ^a^	28.24 ± 0.16 ^b^	24 ± 3 ^c^

For each parameter, different small letter in superscript within row indicates significant differences between samples (*p* < 0.05) comparing samples in mixtures or extrudates. For each sample and parameter, different capital letter in superscript within row indicates significant differences between samples (*p* < 0.05) comparing mixtures and extrudates. Samples were mixtures (M) and extrudates (E); C: Control, 5RHCo: 5% of rosehip co-product and 10RHCo: 10% of rosehip co-product. L* (lightness), a* (red/green coordinate), b* (yellow/blue coordinate), C* (chroma), and h* (tone).

**Table 6:** Phenolic acids (µg/g_dry weight_) of corn mixtures and extrudates. Mean values ± standard deviations.

Phenolic	Mixtures			Extrudates		
Acid	CM	5RHCoM	10RHCoM	CE	5RHCoE	10RHCoE
Caffeic acid	20.72 ± 0.06 ^aA^	18.53 ± 0.12 ^bA^	17.1 ± 0.4 ^cB^	18.3 ± 0.2 ^aB^	18.54 ± 0.19 ^aA^	17.70 ± 0.012 ^bA^
Siringic acid	14.18 ± 0.07 ^aA^	13.7 ± 0.7 ^abA^	12.3 ± 0.4 ^bA^	12.32 ± 0.04 ^aB^	12.1 ± 0.4 ^abB^	11.41 ± 0.08 ^bB^
p-Coumaric acid	64.4 ± 0.2 ^aA^	63.0 ± 0.9 ^aA^	59.4 ± 0.8 ^bB^	59.81 ± 0.05 ^aB^	57.8 ± 0.7 ^bB^	55.8 ± 0.7 ^cA^
Ferulic acid	42.62 ± 0.12 ^aA^	31.38 ± 0.16 ^bB^	29.1 ± 0.6 ^cB^	30.84 ± 0.04 ^cB^	37.7 ± 0.2 ^aA^	36.58 ± 0.03 ^bA^
Di-caffeic	58.28 ± 0.06 ^aA^	45.88 ± 0.19 ^bB^	41.45 ± 0.18 ^cB^	44.4 ± 0.4 ^bB^	53.5 ± 0.7 ^aA^	51.7 ± 0.7 ^aA^
Total	200.18 ± 0.13 ^aA^	172.5 ± 2.1 ^bB^	159.4 ± 0.5 ^cB^	165.63 ± 0.112 ^cB^	180 ± 2 ^aA^	173.2 ± 1.3 ^bA^

For each parameter, different small letter in superscript within row indicates significant differences between samples (*p* < 0.05) comparing samples in mixtures or extrudates. For each sample and parameter, a different capital letter in superscript within row indicates significant differences between samples (*p* < 0.05) comparing mixtures and extrudates. Samples were mixtures (M) and extrudates (E); C: Control, 5RHCo: 5% of rosehip co-product and 10RHCo: 10% of rosehip co-product.

**Table 7:** Hydroxybenzoic acid (Di-Gallic acid) and flavanols content (µg/g_dry weight_) of corn mixtures and extrudates. Mean values ± standard deviations.

	Mixtures			Extrudates		
	CM	5RHCoM	10RHCoM	CE	5RHCoE	10RHCoE
Di-Gall	n.d. ^c^	69.7 ± 0.6 ^bA^	193.2 ± 0.8 ^aA^	n.d. ^c^	38.1 ± 0.7 ^bA^	166.3 ± 0.6 ^aB^
Procyan d1	n.d. ^c^	92.8± 0.4 ^bA^	143.1 ± 0.8 ^aA^	n.d. ^c^	34.7 ± 0.5 ^bB^	62.0 ± 0.7 ^aB^
Procyan d2	n.d. ^c^	32.7 ± 0.4 ^bA^	169.4 ± 0.5 ^aA^	n.d. ^c^	11.8 ± 0.4 ^bB^	71.8 ± 0.5 ^aB^
Cat	n.d. ^c^	197.8 ± 0.3 ^bA^	361.2 ± 1.2 ^aA^	n.d. ^c^	79.0 ± 1.2 ^bB^	283.7 ± 0.6 ^aB^
Q-acet-rham	n.d. ^c^	32.7 ± 1.3 ^bA^	60.0 ± 0.9 ^aA^	n.d. ^c^	19.3 ± 0.3 ^bB^	23.9 ± 0.2 ^aB^
I-glucur	n.d. ^c^	32.9 ± 0.2 ^bA^	75.7 ± 0.98 ^aA^	n.d. ^c^	17.3 ± 0.6 ^bB^	40.4 ± 0.3 ^aB^
Q-gluc	n.d. ^c^	20.77 ± 0.06 ^bA^	48.98 ± 0.98 ^aA^	n.d. ^c^	15.52 ± 0.3 ^bB^	37.3 ± 0.2 ^aB^
Q-glu-gluc-rham	n.d. ^c^	20.6 ± 0.3 ^bA^	30.39 ± 0.14 ^aA^	n.d. ^c^	16.4 ± 0.2 ^bB^	25.99 ± 0.12 ^aB^
I-gluc	n.d. ^c^	23.6 ± 0.4 ^bA^	34.0 ± 0.7 ^aA^	n.d. ^c^	19.3 ± 0.4 ^bB^	29.11 ± 0.13 ^aB^
I-acet-gluc-gluc	n.d. ^c^	24.09 ± 0.16 ^bA^	33.63 ± 0.15 ^aA^	n.d. ^c^	19.8 ± 0.2 ^bB^	31.0 ± 0.7 ^aB^
Q	n.d. ^c^	11.3 ± 0.4 ^bA^	29.0 ± 0.5 ^aA^	n.d. ^c^	8.69 ± 0.04 ^bB^	23.7 ± 0.4 ^aB^
Total	n.d. ^c^	503 ± 4 ^bA^	975 ± 5 ^aA^	n.d. ^c^	280 ± 4 ^bB^	694 ± 3 ^aB^

For each parameter, different small letter in superscript within row indicates significant differences between samples (*p* < 0.05) comparing samples in mixtures or extrudates. For each sample and parameter, a different capital letter in superscript within row indicates significant differences between samples (*p* < 0.05) comparing mixtures and extrudates. Samples were mixtures (M) and extrudates (E); C: Control, 5RHCo: 5% of rosehip co-product and 10RHCo: 10% of rosehip co-product; Di-Gall: Di-Gallic acid; Procyan d1: Procyanidin dimmer 1; Procyan d2: Procyanidin dimmer2; Cat: Catechin; Q-acet-rham: Quercetin-acetyl-rhamnoside; I-glucur: Isorhamnetin-glucuronide; Q-gluc: Quercetin-glucoside; Q-glu-gluc-rham: Quercetin-glucosylglucosyl-rhamnoside; I-gluc: Isorhamnetin-glucoside; I-acet-gluc-gluc: Isorhamnetin-acetyl-glucosyl-glucoside; Q: Quercetin; n.d.–not detected.

**Table 8:** Carotenoids content (µg/g_dry weight_) of corn mixtures and extrudates. Mean values ± standard deviations.

	Mixtures			Extrudates		
Carotenoids	CM	5RHCoM	10RHCoM	CE	5RHCoE	10RHCoE
Lutein	1.50 ± 0.02 ^cA^	1.96 ± 0.04 ^bA^	2.16 ± 0.06 ^aA^	0.39 ± 0.03 ^bB^	0.73 ± 0.3 ^bB^	1.34 ± 0.24 ^aB^
Zeaxanthin	3.49 ± 0.06 ^cA^	4.28 ± 0.10 ^bA^	5.07 ± 0.16 ^aA^	0.59 ± 0.3 ^bA^	0.83 ± 0.14 ^bB^	1.2 ± 0.11 ^aB^
Lycopene	0.42 ± 0.02 ^cA^	5.91 ± 0.25 ^bA^	9.90 ± 0.19 ^aA^	0.14 ± 0.02 ^cB^	1.82 ± 0.33 ^bB^	3.47 ± 0.50 ^aB^
β Carotene	0.46 ± 0.04 ^cA^	4.86 ± 0.18 ^bA^	8.78 ± 0.24 ^aA^	0.16 ± 0.04 ^cB^	1.48 ± 0.19 ^bB^	2.77 ± 0.33 ^aB^
Zea-ester	0.28 ± 0.05 ^cA^	3.67 ± 0.22 ^bA^	6.43 ± 0.14 ^aA^	0.12 ± 0.02 ^cB^	0.90 ± 0.07 ^bB^	1.92 ± 0.08 ^aB^
Lut-ester	0.25 ± 0.02 ^cA^	1.22 ± 0.17 ^bA^	1.95 ± 0.05 ^aA^	0.21 ± 0.02 ^cA^	0.47 ± 0.07 ^bB^	0.70 ± 0.09 ^aB^
Total	6.44 ± 0.23 ^cB^	21.93 ± 1.00 ^bA^	34.3 ± 0.9 ^aA^	1.63 ± 0.12 ^cB^	6.25 ± 0.80 ^bB^	11.4 ± 1.4 ^aB^

For each parameter, different small letter in superscript within row indicates significant differences between samples (*p* < 0.05) comparing samples in mixtures or extrudates. For each sample and parameter, different capital letter in superscript within row indicates significant differences between samples (*p* < 0.05) comparing mixtures and extrudates. Samples were mixtures (M) and extrudates (E); C: Control, 5RHCo: 5% of rosehip co-product and 10RHCo: 10% of rosehip co-product; Zea-ester: Zeaxanthin-ester; Lut-ester: Lutein-ester.

**Table 9:** Ascorbic acid (AA), dehydroascorbic acid (DHAA), vitamin C, folates and antioxidant capacity (AC) content (µg/g_dry weight_) of corn mixtures and extrudates. Mean values ± standard deviations.

	Mixtures			Extrudates		
	CM	5RHCoM	10RHCoM	CE	5RHCoE	10RHCoE
AA	77.3 ± 0.44 ^cA^	217.1 ± 0.72 ^bA^	350.5± 0.8 ^aA^	33.3 ± 0.4 ^cA^	198.3 ± 0.7 ^bB^	318.6 ± 0.2 ^aB^
DHAA	125.88 ± 0.36 ^cA^	175.94.1 ± 0.4 ^bA^	181.1 ± 0.5 ^aA^	22.88 ± 0.12 ^cA^	135.5 ± 0.6 ^bB^	138.1 ± 0.3 ^aB^
Vitamin C	203.2 ± 0.8 ^cA^	393.05 ± 1.13 ^bA^	531.6 ± 1.3 ^aA^	56.2 ± 0.5 ^cA^	333.84 ± 1.3 ^bB^	456.7 ± 0.5 ^aB^
Folates	0.79 ± 0.03 ^cA^	11.04 ± 0.3 ^bA^	17.2 ± 0.6 ^aA^	0.72 ± 0.03 ^cA^	10.11 ± 0.14 ^bA^	15.1 ± 0.7 ^aA^
AC (TEq)	124 ± 18) ^cA^	799 ± 18 ^bA^	1284 ± 14 ^aA^	106 ± 2 ^cB^	352 ± 8 ^bB^	396 ± 9 ^aB^

For each parameter, different small letter in superscript within row indicates significant differences between samples (*p* < 0.05) comparing samples in mixtures or extrudates. For each sample and parameter, a different capital letter in superscript within row indicates significant differences between samples (*p* < 0.05) comparing mixtures and extrudates. Samples were mixtures (M) and extrudates (E); C: Control, 5RHCo: 5% of rosehip co-product and 10RHCo: 10% of rosehip co-product.
